# Enhancing the Analysis of Rheological Behavior in Clinker-Aided Cementitious Systems Through Large Language Model-Based Synthetic Data Generation

**DOI:** 10.3390/ma18153579

**Published:** 2025-07-30

**Authors:** Murat Eser, Yahya Kaya, Ali Mardani, Metin Bilgin, Mehmet Bozdemir

**Affiliations:** 1 Department of Computer Engineering, Bursa Uludag University, Bursa 16059, Turkey; 512131001@ogr.uludag.edu.tr (M.E.); 512231003@ogr.uludag.edu.tr (M.B.); 2 Department of Civil Engineering, Bursa Uludag University, Bursa 16059, Turkey; 512126007@ogr.uludag.edu.tr (Y.K.); alimardani@uludag.edu.tr (A.M.)

**Keywords:** rheological properties, cement admixture compatibility, grinding aids, artificial intelligence, supervised learning, large language models

## Abstract

This study investigates the parameters influencing the compatibility between cement and polycarboxylate ether (PCE) admixtures in cements produced with various types and dosages of grinding aids (GAs). A total of 29 cement types (including a control) were prepared using seven different GAs at four dosage levels, and 87 paste mixtures were produced with three PCE dosages. Rheological behavior was evaluated via the Herschel–Bulkley model, focusing on dynamic yield stress (DYS) and viscosity. The data were modeled using CNN, Random Forest (RF), and Neural Classification and Regression Tree (NCART), and each model was enhanced with synthetic data generated by Large Language Models (LLMs), resulting in CNN-LLM, RF-LLM, and NCART-LLM variants. All six variants were evaluated using R-squared, Mean Absolute Error (MAE), Root Mean Square Error (RMSE), and Logcosh. This study is among the first to use LLMs for synthetic data augmentation. It augmented the experimental dataset synthetically and analyzed the effects on the study results. Among the baseline methods, NCART achieved the best performance for both viscosity (MAE = 1.04, RMSE = 1.33, R^2^ = 0.84, Logcosh = 0.57) and DYS (MAE = 8.73, RMSE = 11.50, R^2^ = 0.77, Logcosh = 8.09). Among baseline models, NCART performed best, while LLM augmentation significantly improved all models’ predictive accuracy. It was also observed that cements produced with GA exhibited higher DYS and viscosity than the control, likely due to finer particle size distribution. Overall, the study highlights the potential of LLM-based synthetic augmentation in modeling cement admixture compatibility.

## 1. Introduction

Greenhouse gas emissions, air pollution, and climate change are among the leading causes of global problems. In addressing these issues, developing alternatives to products that produce carbon dioxide (CO_2_) emissions during production, as well as renewable energy sources and energy efficiency, have become increasingly important [1,2].

Cement is the building block of the modern world, but the large amount of energy and natural resources used in its production pose a serious burden on our planet. More than 2% of the electricity used worldwide and 5% of the electricity used in industry is spent on cement production [3]. This significantly increases the amount of carbon dioxide in the atmosphere, accelerating climate change. In fact, each ton of cement production causes approximately one ton of CO_2_ emissions [2,4,5]. This shows that the cement industry accounts for a significant portion of global carbon emissions. Therefore, urgent steps must be taken to develop more sustainable production methods and reduce the environmental impact of cement production.

Approximately 35% of the energy consumed in cement production is spent in the clinker grinding stage. Furthermore, a significant portion of the energy expended in the grinding process is wasted as heat, sound, and vibration. As a result of studies conducted to reduce both energy consumption and costs, as well as to decrease the amount of CO_2_ released into the environment, the use of grinding aids (GAs) has come to the fore [6,7].

GAs are organic or inorganic compounds used to reduce energy consumption and improve cement properties in the cement production process. These substances adsorb on the surface of cement particles thanks to the highly polar functional groups they contain, such as -OH, -NH_2_, and -COOR. Adsorption occurs as a result of interactions such as neutralization of surface charges, van der Waals forces, and hydrogen bonds. In this way, friction between particles is reduced, grinding energy is reduced, and cement particles exhibit a more homogeneous distribution. The use of GA can accelerate hydration reactions by reducing the surface energy of cement and improve cement–admixture compatibility [2,8,9]. It can also affect properties such as fluidity, setting time, and strength of cement. When choosing GA, both grinding efficiency and environmental effects should be considered [10,11]. The use of GA is important in terms of environmental sustainability.

Plank et al. [12] emphasized that GAs and water-reducing admixtures (PCE) have a similar mechanism in terms of adsorption onto the cement grain. Therefore, PCE adhesion to the cement grain may be negatively affected in cementitious systems prepared with cements produced using GA. It has been stated that in case of binder–admixture incompatibility, negative effects such as rapid or slow setting [13], segregation [14], loss of consistency, and increased shrinkage [15] may occur in cementitious systems. Some of these negative effects may be due to binder properties, while some may be due to additive properties. Since the PCEs used affect the cement properties, the cement–PCE compatibility of the cements in which PCEs are used during production should be examined comprehensively. Depending on the type, dosage, and chemical content of the GA used, its effects on both cement surface properties and hydration reactions vary [16]. Some studies examining the compatibility of cements produced with GA with PCE are summarized below.

Sun et al. [17] investigated the compatibility of cements produced using glycerine type GA at 0.01, 0.02, 0.03, and 0.04 ratios with PCE. It was observed that the flow performance of PCE is higher when the GA dosage is less than 0.02%. It was determined that a lower percentage of PCE was adsorbed on cements containing more than 0.02% GA. In another study, the effect of the use of GA on the surface energy of the cement grain was investigated by Prziwara and Kwade [11]. It was stated that GA adsorbed onto the cement grain, causing a decrease in surface energy. On the other hand, the cements produced using GA may physically have higher surface energy compared to the grains without GA due to the smaller particles. This situation revealed that there are different opinions about the adsorption of PCE to cements containing GA. It was stated that PCE adsorption was seriously affected by the high positive charge density of the cement surface [18,19]. Sun et al. [17] emphasized that the cement surface charge density can vary depending on the type of GA and its usage rate. Accordingly, the degree of adsorption of PCE to cement varies in cements containing GA. As can be understood from the literature, the PCE compatibility of cemented systems should be examined in the presence of GA.

Mardani-Aghabaglou et al. [13] reported that determining the rheological parameters of cementitious systems is one of the most reliable methods for examining cement-admixture compatibility. It was emphasized that many experiments should be performed to determine the parameters in question [20]. Many modeling and regression studies were carried out to facilitate this very laborious process [21]. Artificial neural networks and regression models are widely applied for this purpose [21]. In their study, Venkata Rao et al. [22] used different approaches to predict the shear behavior of glass fiber reinforced concrete slabs. They adopted artificial neural network modeling in their study and used Mean Absolute Error and Root Mean Square Error criteria. Revathi et al. [23] conducted modeling studies to alleviate the experimental load of geopolymer mixtures for sustainable production. In this direction, they used machine learning techniques effectively in mixture design and interpretation. In another study, Nagaraju et al. [24] estimated the compressive strength of concrete mixtures with the help of linear regression and Bayesian optimization. The compressive strength of concrete was determined by multivariate adaptive regression splines (MARS), artificial colony, and artificial neural networks (ANNs) by Cheng and Cao [25]. In another study, the compressive strength of concrete was modeled by multiple summation regression trees (MARTs) and ANN methods [26]. Kaveh et al. [27] modeled the compressive strength and fresh state properties of cementitious systems using the M5 Tree and MARS methods. Asteris and Mokos [28] modeled the compressive strength and elasticity modulus of cementitious systems with MARS, M5 Tree model, and ANN, and compared the results. Compressive strength of cementitious systems [28,29,30,31] flexural strength [32,33], V-funnel flow time [31], L-box [27], and durability performance [34,35] were modeled using many methods. It has been understood from the literature that many studies were conducted to model the strength and durability properties of cementitious systems. However, a limited number of studies were carried out on the modeling of rheological parameters. Mardani-Aghabaglou et al. [36] investigated the compatibility of different types of cements with water-reducing admixture by modeling them with ANN, MARS, and CRA methods. No study was found in which the rheological parameters of cementitious systems containing GA were modeled. It has been determined that there is a limited number of studies in the literature examining and modeling the cement–water reducing admixture compatibility of cements produced with GA. In this study, the rheological properties, such as dynamic yield shear stress and final viscosity of cement paste mixtures containing GA, were modeled, and the effective parameters were determined according to their order of action. For this purpose, Neural Classification and Regression Tree (NCART), Random Forest (RF), and Convolutional Neural Network (CNN) methods were applied. In particular, the ncart method has been recently introduced to the literature and stands out due to its high performance in cases where the dataset is limited and because it has not been used before in the estimation of DYS and viscosity values. In addition, synthetic data was generated using Large Language Models to improve model performance, and the effects on the models were examined. Mean Absolute Error (MAE), Root Mean Square Error (RMSE), R-squared, and Logcosh metrics were used to measure the performance of the models.

## 2. Experimental Study

In the modeling study, data obtained from the authors’ previous experimental study [3,37] were used. The properties and test methodology of the materials used in the study are given below.

### 2.1. Materials

**Figure 1:**
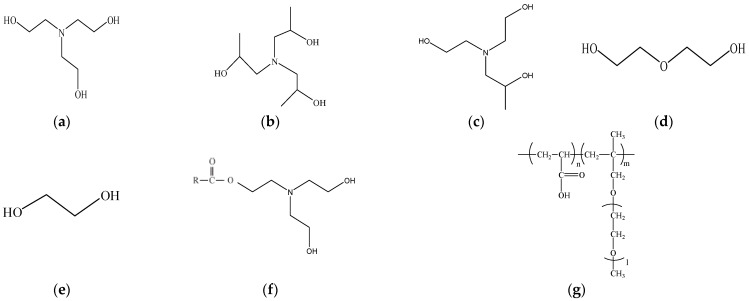
(**a**) TEA, (**b**) TIPA, (**c**) DEIPA, (**d**) DEG, (**e**) EG, (**f**) M-TEA-1-2, (**g**) PCE [39].

The cements used in the study were obtained by grinding 4.8 kg of clinker and 0.2 kg of gypsum (gypsum at s rate of 4% of the total ground mass) in a laboratory-type mill. In addition to 5 different GAs that are widely used in the industry, 2 modified GAs—obtained as a result of the modification of the TEA additive through the esterification reaction— were also included, resulting in a total of 7 GAs used. The GAs were used in four different ratios, 0.025%, 0.05%, 0.075%, and 0.1% of the total weight of clinker and gypsum during the clinker grinding phase. Thus, in addition to the control cement that does not contain GA, a total of 29 different CEM I 42.5R type cements in compliance with the EN 197-1 Standard [38] were produced. All cements were ground to a target Blaine fineness of 4100 ± 100 cm^2^/g. The Blaine fineness value and particle size distributions of the cements used are shown in Table 1. A uniform polycarboxylate ether-based high-rate water-reducing admixture (PCE) was used. Some properties of the used GA and PCE provided by the manufacturer are shown in Table 2, and their molecular representations are shown in Figure 1.

### 2.2. Preparation of Mixtures

In order to determine the rheological parameters, 87 paste mixes with 0.32 *w*/*c* ratio and 0, 0.1, and 0.15% PCE by cement weight were prepared. In the selection of these ratios, the measuring capacity of the rheometer used and the mixing capacity of the mixer used were taken into account. The naming of the paste mixtures was made according to the type of GA used and its rate of use. For example, the mixture without GA is denoted by C, while the mixture containing 0.025% TIPA type GA is named 0.025 TIPA.

### 2.3. Method

The rheological parameters of the mixtures were measured with the help of the Herschel–Bulkley model shown in Equation (1) using a ball measuring system–BMS rheometer. Similar measurement systems were applied by other researchers [2,40,41,42]

The relationship between shear stress (*τ*) and shear rate $\overset{\cdot }{\gamma }=\left(\frac{d\gamma }{dt}\right)$ according to the Herschel–Buckley model is shown in Equation (1).

(1) $$\tau =\tau o+b\cdot {\dot{\gamma }}^{\rho }$$

Here, τ is the dynamic shear stress, τ_o_ is the dynamic threshold shear stress, b is the consistency coefficient, p is the Herschel–Bulkley index, and $\overset{\cdot }{\gamma }$ = (dγ/dt) is the deformation rate.

## 3. Modeling Methodology

The rheological properties (DYS and viscosity) of paste mixtures prepared using GA and control cement without GA are shown in Table 3. Three different PCE ratios were used to examine the compatibility of the cements with the water-reducing admixture. Cement–PCE compatibility was examined based on the changes in these rheological parameters.

Two different methods were used in modeling the data: hybrid learning, machine learning, and deep learning. Neural Classification and Regression Tree (NCART) was used as a hybrid learning method, Random Forest (RF) was used as the learning method, and Convolutional Neural Network (CNN) was used as the deep learning method. Four different metrics were used to evaluate the results: Mean Absolute Error (MAE), Root Mean Square Error (RMSE), R-squared, and Logcosh.

**Figure 2:**
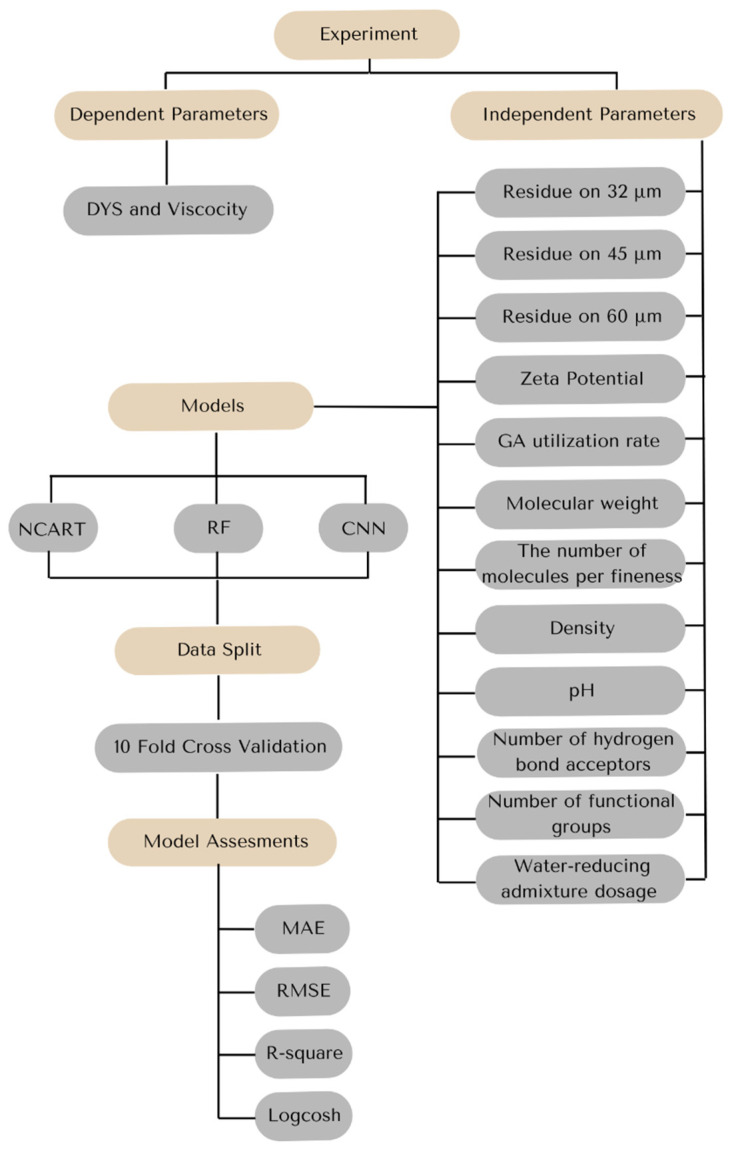
Regression modeling flowchart for clinker-aided cementitious rheology.

Cross-validation is a statistical method used to evaluate the predictive performance of a model by dividing the original dataset into multiple subsets, training the model on some of them, and validating it on the remaining data. This approach is especially useful in cases where data is limited, as it maximizes the use of the dataset while reducing the risk of overfitting [43,44,45]. One of the most commonly used cross-validation techniques is k-fold cross-validation, where the dataset is divided into k equal parts. The model is trained on k-1 folds and tested on the remaining folds; this process is repeated k times, so that each fold serves as a validation set once [45,46]. In the study, the 10-fold cross-validation technique was used to avoid the memorization problem due to the low number of data points and to create robust models. The general methodology followed in this study is presented in Figure 2.

Python programming language version 3.9 was used in the modeling processes. The Keras library was preferred for the implementation of the CNN model. While obtaining the results of the NCART method, 5000 epochs were run, for the n_trees parameter 2–10, for the n_layers parameter 2–10, and for the n_selected parameter 4–6. While obtaining the results of the NCART method, 5000 epochs were run, for the n_trees parameter 2–10, for the n_layers parameter 2–10, and for the n_selected parameter 4–6. As a result of setting the parameters for the DYS experiment, n_trees: 2, n_layers: 4, n_selected: 4, and for the viscosity experiment, n_trees: 10, n_layers: 4, n_selected: 6. For CNN, the average values of the metrics obtained after 5000 iterations were calculated. In the CNN method, Sigmoid and Linear activation functions were used for the hidden layer and output layer, respectively. Optimization was performed between 3 and 199 trees while deciding how many decision trees would be used for the RF algorithm. The depth value that produced the best results was selected, and the operations were performed. According to the results of the optimization process, the depth values for the viscosity and dynamic yield stress (DYS) parameters were found to be 19 and 61, respectively. Equation (2) was used in the Linear Regression Analysis (LR) method to determine the coefficients within the scope of the study. The aim of determining the coefficients is for researchers to obtain output with their data.

(2) $$\sum_{i=1}^{M}{\left(y_{i}-\underline{y_{i}}\right)}^{2}=\sum_{i=1}^{M}{\left(y_{i}-\sum_{j=0}^{p}w_{j}\times x_{ij}\right)}^{2}$$

### 3.1. Neural Classification and Regression Tree (NCART)

NCART, a new model for analyzing tabular data, combines the advantages of deep learning and decision tree [47]. NCART combines the computational power of deep learning models and the interpretability of decision trees to create effective and successful prediction models. During the implementation of the method, 5000 training time, 2–10 as the number of decision trees, 2–10 as the neural network layers, and 4–6 value ranges for the n_selected parameter were used.

### 3.2. Random Forest (RF)

It was stated that the RF method is one of the most widely used decision tree-based ensemble learning methods, which produces multiple classifiers instead of a classifier and then classifies the test data with the votes obtained from the estimates [48].

Each dataset was generated from the original dataset by displacement, then the trees were developed using random feature selection [49]. It was reported that the RF method is very fast and has high accuracy [50,51].

### 3.3. Convolutional Neural Network (CNN)

Convolution layer and neuron numbers were applied as 1–2 and 64, 128, 16–8, 32–16, respectively. The hidden layer numbers are 2 and 3; the number of neurons in the hidden layers is realized as 32–16; 16–8–4; 32–16–8. The learning rate and momentum coefficients were taken as 0.01 and 0, respectively.

### 3.4. LLMs (Large Language Models)

Large Language Models (LLMs) are artificial intelligence systems designed to generate human-like text. Their foundations trace back to the 2017 paper “Attention Is All You Need” [52], which marked a pivotal milestone in natural language processing; the Transformer architecture introduced therein has become the cornerstone of contemporary large-scale language models. By leveraging LLMs, a wide array of tasks—such as text comprehension, text generation, machine translation, information retrieval, information extraction, and even synthetic dataset creation—can be performed with high efficiency. GPT-4o was used for synthetic data augmentation in the study [53].

### 3.5. Evaluation of Model Performance

In order to evaluate the results of the 3 modeling methods applied in the study, 3 different metrics, namely MAE, RMSE, R-squared, and Logcosh, presented in Equations (3)–(6), were used. Based on the statistical parameters used in Equations (3)–(6), lower MAE, RMSE, and Logcosh and higher R-squared values are the desired outcomes.

(3) $$MAE=\frac{1}{n}\sum_{i=1}^{n}\left|E_{i}-P_{i}\right|$$

(4) $$RMSE={\left[\frac{1}{n}\sum_{i=1}^{n}{\left(E_{i}-P_{i}\right)}^{2}\right]}^{1/2}$$

(5) $$R-squared=1-\frac{\sum {\left(E_{i}-E_{p}\right)}^{2}}{\sum {\left(E_{i}-Mean\right)}^{2}}$$

(6) $$Logcosh=\sum_{i=1}^{n}log(cosh(P_{i}-E_{i}))\;cosh(x)=\frac{e^{x}+e^{-x}}{2}$$

## 4. Results and Discussion

When the results in Table 3 were examined, the yield stress and viscosity values generally decreased with the addition of PCE to the mixture, as expected. This behavior became more evident with the increase in PCE dosage. DYS and final viscosity values of the mixtures generally increased with the increase in GA dosage. As GAs are adsorbed on the cement particle, they reduce agglomeration and provide fluidity [2,17]. In the case of low-dosage GA use, reductions in the rheological values of paste mixtures were observed depending on the mentioned mechanism. However, the adsorption of PCE on the particle surface decreases when the surface of the cement particles is surrounded by the GA in the case of high GA. It is understood from Table 1 that as the GA dosage increases, the fine particle ratio increases in cements having GA with the same Blaine fineness value as the control cement. Obtaining finer cements by using GA has both positive and negative effects. While finer grains increase water demand and negatively affect rheological parameters, they can suppress this negative effect by causing higher PCE adsorption. Sun et al. [17] reported that GA can adsorb more strongly to finer particles. It is thought that PCE adsorption is lower due to the higher GA adsorption in finer particles. As the adsorption of PCE to the cement particle increases, the dispersion ability of the admixture may also increase [18]. Moreover, a high dosage of GAs may reduce PCE adsorption due to the competitive adsorption of GAs [9,19,54]. Under such conditions, increasing the PCE dosage in the presence of elevated GA levels results in a higher concentration of non-adsorbed PCE within the system. This may cause the main and side chains of non-adsorbed PCE molecules to become entangled and aggregate [39,42,55]. On the other hand, depending on the formation of smoother particles in the presence of GA, it can positively affect the rheological properties. This observation underlines the need for accurate modeling of material behavior in systems affected by additive interactions. In a related study, Ai et al. [56] proposed a numerical model to simulate physical phenomena such as melt pool and keyhole evolution, demonstrating that detailed modeling can enhance the predictive capability in complex material systems.

It has been understood that while the positive effects of GA are dominant with the use of GA at a lower dosage (0.025), the negative effects come to the fore as the GA dosage increases.

The mixtures containing 0.025% and 0.05% GA showed the lowest performance in terms of the rheological properties (high DYS and FV of the TEA mixture). The lowest performance was measured in the DEIPA mixture when the GA content was above 0.05%. Mixtures containing M-TEA-2 exhibited the highest rheological performance (low DYS and FV values), regardless of the GA usage rate.

### 4.1. Determining the Dominant Parameters

**Figure 3:**
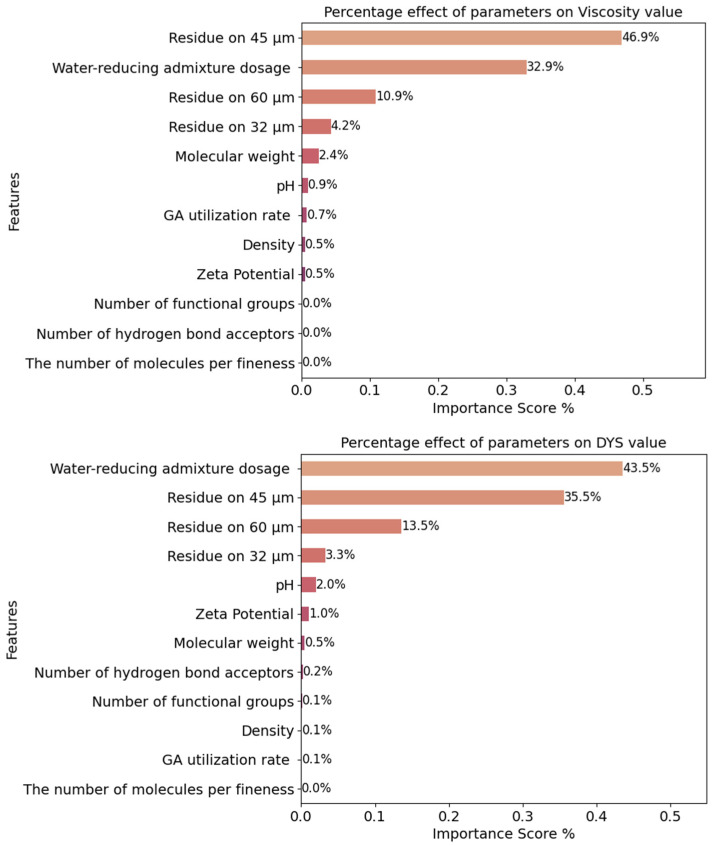
Dominant value ranking affecting viscosity and DYS predictions by the RF method.

The RF method was used to determine the dominant parameters affecting the rheological properties. For this purpose, the feature importance method was taken into account. The ranking obtained for the dominant parameters from the RF model for viscosity and DYS data is shown in Figure 3.

Figure 3 shows the percentage of the independent variables affecting the values of the dependent variables DYS and viscosity in such a way that the total values are 100%. In terms of viscosity characteristics, the dominant parameters were determined, respectively, as follows:

The material amount residue on 45 microns, water-reducing admixture dosage, the material amount residue on 60 microns, the material amount residue on 32 microns, zeta potential, molecular weight, pH, GA utilization rate, density, the number of hydrogen bond acceptors, the number of functional groups, number of molecules per fineness.

Water-reducing admixture dosage, the material amount residue on 45 microns, the material amount residue on 32 microns, the material amount residue on 60 microns, pH, zeta potential, molecular weight, GA utilization rate, density, the number of functional groups, the number of hydrogen bond acceptors, the number of molecules per fineness. The mentioned ranking for DYS was as follows:

As expected, water reducing admixture dosage and particle size distribution were the most dominant parameters in terms of rheological properties. In this context, the cement particle size distribution parameter was more dominant than the Blaine fineness value. It was previously emphasized that cement particle size distribution was directly affected by GA type and usage rate. Therefore, rheological parameters of cementitious systems were affected by the presence of GA. In addition to the positive effects of GA usage in terms of energy efficiency, it should be considered that it may also provide benefits in terms of rheological properties and cement-PCE compatibility of cementitious systems.

**Figure 4:**
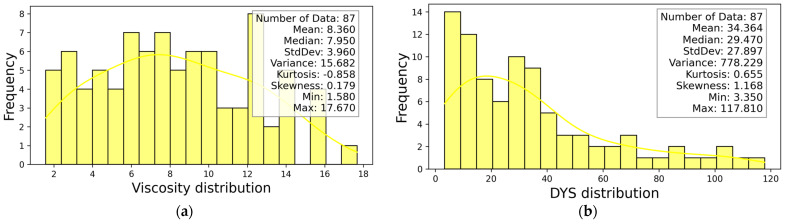
Distributions of experimental values (**a**) Viscosity, (**b**) DYS.

**Figure 5:**
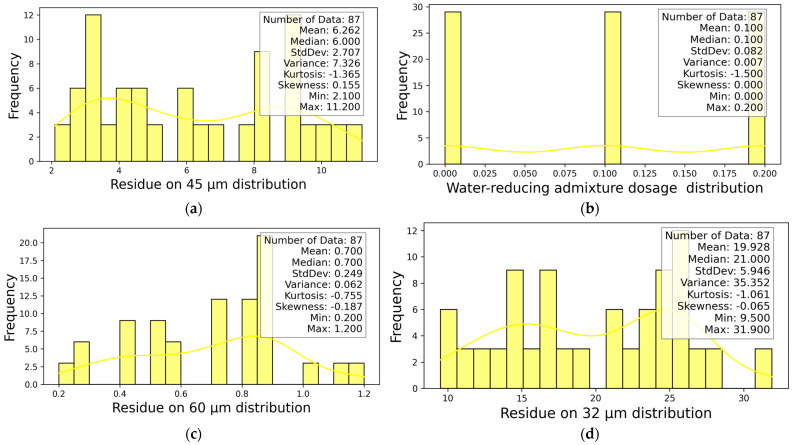
The four most effective parameters for distribution (**a**) Residue on 45 µm, (**b**) Water-reducing admixture dosage, (**c**) Residue on 60 µm, (**d**) Residue on 32 µm.

The statistical information of DYS and viscosity-dependent variables is presented in Figure 4. After determining the dominant parameters affecting the DYS and viscosity-independent variables, the statistical information of the four most effective independent parameters is presented in Figure 5.

### 4.2. Factors Affecting Cement–Admixture Compatibility

#### 4.2.1. Cement Fineness and Its Role in Cement–PCE Compatibility

Cement fineness significantly influences compatibility with water-reducing admixtures [18]. Both Blaine fineness and particle size distribution impact surface energy and adsorption capacity [57,58]. Finer particles (<10 µm) enhance PCE adsorption due to higher surface energies [17,36]. Additionally, the presence of GAs alters surface energies, adsorption capacities, and Zeta potential, affecting the compatibility of GAs with PCEs [2].

#### 4.2.2. Grinding Aid Properties and Their Effect on Cement Rheology

The performance of GAs depends on parameters such as pH, molecular weight, utilization rate, and functional groups [11]. Comparisons based on molecular count rather than mass provide better insight into adsorption performance. GAs with pH values differing from neutrality (e.g., TEA, TIPA) enhance rheological properties [2,11]. These findings align with earlier studies on the impact of pH on GA performance [2].

#### 4.2.3. Structural and Chemical Factors of PCEs Affecting Cement Rheology

PCE characteristics such as backbone length, side chain number, molecular weight, and intermolecular bonds significantly influence dispersion and rheological parameters [58,59,60,61]. Comb-like side chains of PCEs enhance dispersion compared to GAs [2], and the utilization rate remains the most critical factor for improving rheological properties [36].

### 4.3. Dataset Power Analysis

When performing power analyses on the dataset, power calculations were performed using Cohen’s F^2^ effect size given in Equation (7) and the F-test statistic given in Equation (8). The equation shows the R^2^ variance ratio, k predictor variable count, and n sample count.

(7) $$f^{2}=\frac{R^{2}}{1-R^{2}}$$

(8) $$F=\frac{\frac{R^{2}}{k}}{\frac{1-R^{2}}{n-k-1}}$$

According to the power analysis evaluations, the dataset used in the study was found to be sufficient, with a power value of 0.956 in terms of a large effect. The small and medium effect values are 0.096 and 0.573, respectively, and it has been calculated that the number of data points needs to be increased. Therefore, synthetic data augmentation was applied using the LLM to improve the reliability of the model in terms of small and medium effects.

## 5. Modeling Results

The coefficients obtained from the regression analysis applied to the DYS and viscosity outputs are summarized in Table 4. The coefficients of the experimental parameters in the viscosity and DYS experiments were calculated by regression analysis methods and presented in Table 4. The weights in Table 4 can be used as in Equation (9). In the table, weights were calculated for 12 different features (inputs) of the experiments. By using the weights of these features, predictions can be generated for data that is not available in the dataset or is available to another researcher. When Equation (9) is examined, the value in the viscosity experiment for the fineness feature (input), denoted by $X_{1},$ is multiplied by the weight coefficient 13.2043. After this process is applied to the remaining 11 features, the intercept value in the last column of the table is added to the result. The result is the output prediction of the data from the related viscosity experiment. Using the weights presented in Table 4, prediction values can be obtained for all data.

(9) $$Prediction=X_{1}\times 13.2043+X_{2}\times (-4.4515)+X_{3}\times (-60.9363)+X_{4}\times 3.9518+X_{5}\times 15.2265+X_{6}\;\times (-7.5471)+X_{7}\times 0.0000+X_{8}\times (-5.1809)+X_{9}\times 14.2919+X_{10}\times 6.1875+X_{11}\times 6.1875+X_{12}\;\times (-43.2357)+65.8933$$

CNN results for viscosity and dynamic yield stress (DYS) are presented in Table 5. To achieve optimal performance, the architecture was systematically varied by adjusting the number of convolutional layers and their neurons, as well as the number of hidden layers and their neuron counts.

As shown in Table 5, the best result for viscosity using the CNN method is obtained with one convolutional layer containing 128 neurons and two hidden layers with 32 and 16 neurons, respectively. Table 5 shows that the best result for DYS in the CNN method is obtained when the number of neurons in one convolutional layer is 128 neurons, and the number of neurons in two hidden layers is 32-16 neurons.

In the viscosity experiment, the CNN model was more accurate in predicting the target value, whereas in the DYS experiment, it performed less well in predicting the target value.

The RF model results are presented in Table 6. As shown, viscosity predictions made with the RF method achieved higher accuracy compared to those for DYS.

Results from the NCART model for viscosity and DYS are shown in Table 7. When the data in Table 7 were examined, the results obtained for viscosity in the NCART method were better than DYS. It is thought that the reason why all models produce better results in the viscosity experiment than in the DYS experiment is that the DYS results are spread over a larger area compared to the viscosity results. The stability of the metric rankings measured using other methods prior to this point is crucial to ensure consistent results.

In order to determine the most successful method for viscosity and DYS properties, the values obtained for all metric types are compared in Table 8, and the values obtained with augmented data are compared in Table 9.

**Figure 6:**
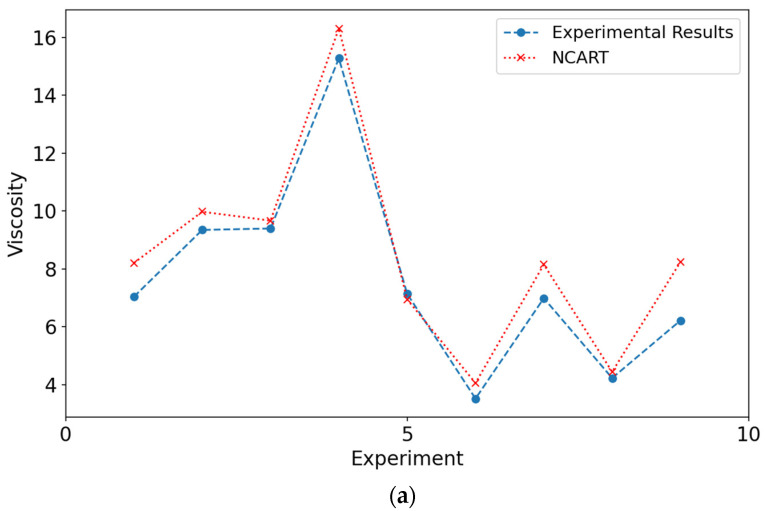

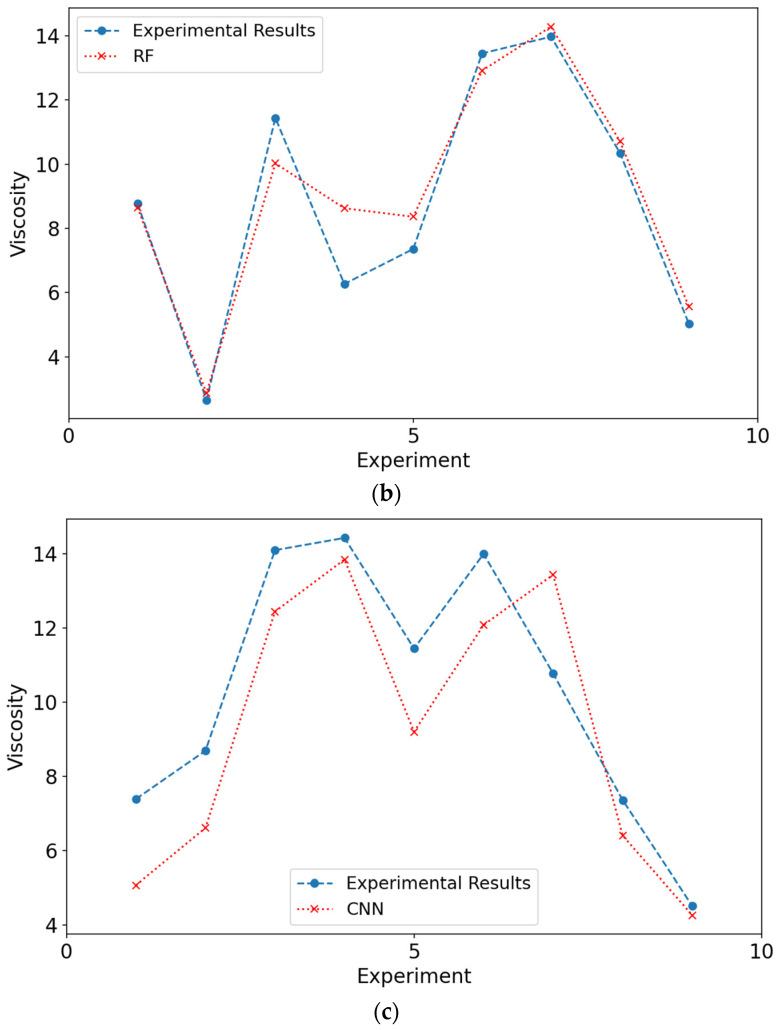
Performance of the models in predicting viscosity values in the test phase: (**a**) Experimental results and NCART, (**b**) experimental results and RF, (**c**) experimental results and CNN.

**Figure 7:**
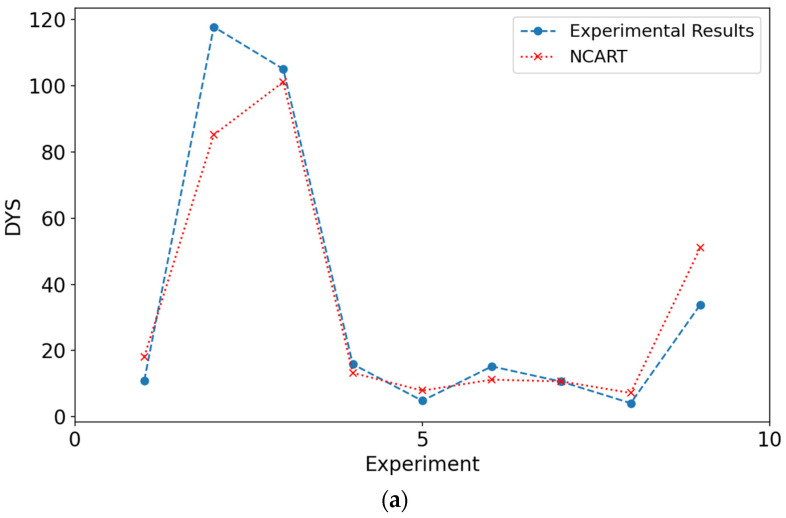

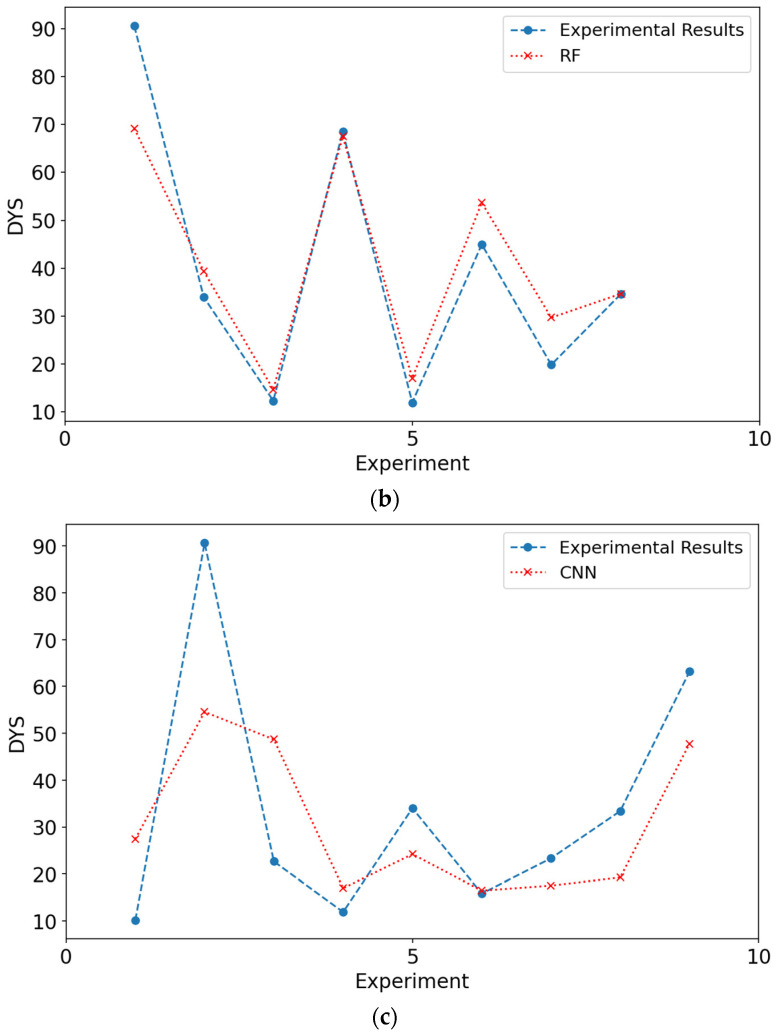
Performance of the models in predicting DYS values in the test phase: (**a**) Experimental results and NCART, (**b**) experimental results and RF, (**c**) experimental results and CNN.

Charts that compare results from experimental data and models are shown in Figure 6 and Figure 7. When the figures for both DYS and viscosity were examined, it was seen that the results obtained from the modeling are close to the experimental values. The closeness of these values to each other can be shown as evidence for the validity of the models. Figure 6 and Figure 7 show the experimental values of viscosity and DYS and the values obtained by the models during testing.

From Table 8, it is seen that the NCART method gives the best results in all metrics for both viscosity and DYS parameters. The RF method gives the second-best results in all metrics for both viscosity and DYS parameters. The CNN method was behind the other two models in both experiments. As a result, in terms of viscosity parameters, the results obtained with the NCART method were 0.19 and 1.36 times better than the RF and CNN methods, respectively. For DYS, it was calculated as 0.24 and 10.29, respectively.

According to Table 9, NCART achieves the highest performance across all metrics for both viscosity and DYS. Furthermore, when the experimental data is augmented with synthetic samples generated by the LLM, all baseline models show significant improvements in all measurement metrics. Specifically, NCART-LLM reduces the viscosity test MAE from 1.04 to 0.53 and the DYS test MAE from 8.73 to 3.29, thus outperforming the original NCART by 0.51 and 5.44 MAE points, respectively.

Similarly, RF-LLM reduced RF’s viscosity MAE from 1.23 to 0.97 and DYS MAE from 8.97 to 6.88, while CNN-LLM reduced CNN’s viscosity MAE from 2.40 to 0.75 and DYS MAE from 19.02 to 3.27. Similarly, for all other metrics, RF-LLM and CNN-LMM show significant improvement in target predictions. In particular, the CNN-LLM shows a performance improvement over the CNN model in all metrics, as expected. This can be explained by the fact that the performance of CNN models improves in direct proportion to the amount of data. The performance of deep learning-based models, such as CNN, is directly related to the amount of data. Therefore, the results of the model trained with 87 examples lag behind those of other models. Deep learning-based models require more data for better performance. Therefore, the limited amount of data has reduced the model’s generalization ability. As a result, it demonstrates the significant benefit of LLM-based data augmentation for improving the generalization capabilities of the models.

Charts comparing the results obtained from the augmented experimental data and models are shown in Figure 7. When the results for viscosity and DYS are examined, it is seen that the results obtained from the modeling are closer to the experimental values than the normal models. The closeness of the predictions made by the models with the augmented experimental data confirms the metric results given in Table 9. The close agreement observed in the viscosity and DYS experiments further validates our modeling approach.

## 6. Conclusions

This study aimed to model the compatibility between cements produced with grinding aids (GAs) and water-reducing admixtures (PCE) and to identify the key parameters affecting rheological behavior.

- The results showed that paste viscosity is predominantly influenced by PCE dosage, particle size distribution (residues on 45, 32, and 60 microns), pH, zeta potential, molecular weight, GA dosage, and several molecular descriptors. Similarly, dynamic yield stress (DYS) is primarily affected by residue on 45 microns, PCE dosage, residue on 60 and 32 microns, zeta potential, molecular weight, and other related parameters.
- It was concluded that particle size distribution is the most critical factor determining cement–PCE compatibility. Since GA usage directly influences fineness characteristics, the type and dosage of GA should be carefully selected. Future research is recommended to focus on the relationship between GA use and cement powder properties in greater detail.
- Among the modeling methods employed, NCART showed the best performance in predicting both viscosity and DYS, followed closely by Random Forest (RF). The models achieved R^2^ values of 0.84 for viscosity and 0.77 for DYS, indicating strong predictive capabilities, though some room for improvement remains.
- The study also highlighted the limitations of small datasets, which may reduce model generalizability and increase the risk of overfitting. However, the decision tree-based models used (NCART and RF) proved advantageous due to their non-parametric nature and ability to handle limited data without distributional assumptions.
- In terms of error metrics, Logcosh yielded the lowest errors across all models, followed by MAE and RMSE. When Large Language Model (LLM)-based data augmentation was applied, all models showed substantial improvement. For example, NCART-LLM reduced the MAE of viscosity from 1.04 to 0.53 and of DYS from 8.73 to 3.29. Similar improvements were observed in RF-LLM and CNN-LLM, with CNN showing the most notable gains, likely due to its higher learning capacity and sensitivity to increased data volume.
- Overall, the integration of LLM-generated synthetic data significantly enhanced model performance, especially for CNN and NCART structures. NCART-LLM achieved an R^2^ value of 0.95 for viscosity prediction, and both NCART-LLM and CNN-LLM achieved R^2^ values of 0.95 for DYS prediction. These findings confirm that LLM-augmented data can successfully strengthen model accuracy and robustness in small to medium-sized datasets.
- LLM-generated examples added to real-world data have significantly improved model accuracy by compensating for the lack of power in medium and small domains.

## Figures and Tables

**Table 1:** Particle size distribution of cements.

Cements	Blaine Fineness (cm^2^/g)	Residue on 32 µm Sieve (%)	Residue on 45 µm Sieve (%)	Residue on 60 µm Sieve (%)
Control	4100	31.9	10.4	1.2
0.025 TEA	4110	26.5	7.6	0.8
0.05 TEA	4115	18	4.7	0.7
0.075 TEA	4100	16.6	4	0.5
0.1 TEA	4090	14	3.1	0.3
0.025 TIPA	4100	24.9	8.2	0.9
0.05 TIPA	4180	19.0	4.9	0.8
0.075 TIPA	4200	11.7	2.9	0.5
0.1 TIPA	4140	9.6	2.1	0.2
0.025 DEIPA	4080	25.8	9.5	0.8
0.05 DEIPA	4090	21.0	5.8	0.7
0.075 DEIPA	4100	15.2	3.2	0.5
0.1 DEIPA	4020	13.2	3.0	0.4
0.025 DEG	4110	23.8	8.2	0.9
0.05 DEG	4140	16.7	6.0	0.7
0.075 DEG	4190	12.5	4.8	0.6
0.1 DEG	4150	9.5	3.1	0.4
0.025 EG	4160	25.4	9.3	0.9
0.05 EG	4190	25.8	8.1	0.9
0.075 EG	4180	22.3	6.2	0.9
0.1 EG	4160	17.3	4.3	0.7
0.025 M-TEA-1	4120	24.6	9	0.9
0.05 M-TEA-1	4070	21	6.9	0.6
0.075 M-TEA-1	4100	14.8	3.5	0.4
0.1 M-TEA-1	4050	14.5	3.4	0.3
0.025 M-TEA-2	4080	28.2	11.2	1.1
0.05 M-TEA-2	4080	26.2	10.2	1
0.075 M-TEA-2	4060	24.4	9	0.9
0.1 M-TEA-2	4050	23.5	9	0.8

**Table 2:** Some chemical properties of Gas [39].

GA Name	Alkaline Content (%) (Na_2_O)	Intensity (g/cm^3^)	Solid Content (%)	Chloride Content (%)	pH 25 °C
TEA	<10	1.095	50.0	<0.1	10.5
TIPA	<10	1.124	50.0	<0.1	10.8
DEIPA	<10	1.079	50.0	<0.1	9.7
DEG	<10	1.118	50.0	<0.1	7.2
EG	<10	1.26	50.0	<0.1	8.2

**Table 3:** Dynamic yield stress and viscosity values of mixtures containing different amounts of PCE.

Mixture	DYS (Pa)			FV (Pa s)		
	PCE Ratio (by Weight of Cement)					
	0%	0.1%	0.15%	0%	0.1%	0.15%
C	38.28	30.01	10.93	38.28	30.01	3.77
0.025 TEA	53.5	25.86	10.16	11.1	8.69	4.63
0.05 TEA	83.76	31.19	14.35	12.6	11.06	6.14
0.075 TEA	90.61	46.99	22.65	12.64	14.09	8.77
0.1 TEA	117.81	96.93	54.34	14.42	15.91	13.72
0.025 TIPA	33.91	15.69	5.31	8.78	5.98	2.66
0.05 TIPA	59.39	28.4	12.25	11.44	9.35	5.42
0.075 TIPA	105.09	68.51	32.31	12.61	13.99	9.4
0.1 TIPA	81.4	106.5	34.5	12.52	17.67	10.77
0.025 DEIPA	27.66	11.89	4.32	6.93	4.69	2.22
0.05 DEIPA	34.01	15.88	6.72	9.45	6.97	3.65
0.075 DEIPA	76.25	44.96	21.47	13.06	11.86	9.11
0.1 DEIPA	86.12	62.94	41.63	15.28	15.28	11.68
0.025 DEG	30.08	15.82	5.13	7.14	5.62	2.43
0.05 DEG	36.38	17.48	4.82	8.95	6.27	3.01
0.075 DEG	55.36	29.59	13.58	10.37	8.81	5.39
0.1 DEG	68.73	33.58	15.2	12.6	10.28	6.36
0.025 EG	24.95	14.34	5.45	7.3	5.6	2.75
0.05 EG	43.82	23.32	7.35	10.33	7.91	3.52
0.075 EG	41.29	23.8	8.36	12.05	7.83	3.85
0.1 EG	52.94	30.55	12.24	12.37	10.26	5.82
0.025 M-TEA-1	29.92	10.6	3.66	6.99	4.47	1.94
0.05 M-TEA-1	33.4	15.87	4.09	9.89	7.36	2.88
0.075 M-TEA-1	68.84	42.47	19.78	12.33	13.45	8.53
0.1 M-TEA-1	105	63.17	26.44	13.97	15.89	10.35
0.025 M-TEA-2	34.56	10.72	7.32	7.95	4.52	1.58
0.05 M-TEA-2	41.53	10.55	3.35	8.83	4.23	1.91
0.075 M-TEA-2	29.47	12.04	3.7	6.68	5.03	1.99
0.1 M-TEA-2	33.85	14.81	5.87	7.71	6.22	3.09

**Table 4:** Coefficients of regression models for viscosity and DYS values.

Experiment	w0	w1	w2	w3	w4	w5	w6	w7	w8	w9	w10	w11	Intercept
Viscosity	0.268	−0.883	−7.029	−0.016	22.494	−0.005	0.000	0.453	0.107	0.670	0.670	−24.778	9.9780
DYS	13.204	−4.451	−60.936	3.9518	15.226	−7.547	0.000	−5.180	14.291	6.187	6.185	−43.235	65.893

**Table 5:** CNN results for viscosity and DYS values.

Experiment	Number of Conv. Layer Neurons	Number of Hidden Layers	Number of Hidden Layer Neurons	MAE		RMSE		R-Squared		Logcosh	
				Train	Test	Train	Test	Train	Test	Train	Test
**Viscosity**	128	2	32-16	1.96	**2.40**	2.43	**2.76**	0.61	0.28	1.41	**1.78**
	128	3	32-16-8	2.01	**2.44**	2.47	**2.85**	0.60	0.24	1.46	**1.85**
	64			2.92	**3.10**	3.50	**3.61**	0.18	0.01	2.32	**2.47**
	32-16	3	16-8-4	3.30	**3.47**	3.95	**4.01**	0.01	0.01	2.68	**2.83**
	16-8		32-16-8	3.31	**3.48**	3.96	**4.02**	0.01	0.01	2.69	**2.84**
**DYS**	128	2	32-16	15.07	**19.02**	19.99	**23.00**	0.47	0.01	14.41	**18.33**
	128	3	32-16-8	20.78	**21.11**	28.35	**26.67**	0.01	0.01	20.12	**20.44**
	64			20.54	**20.92**	28.09	**26.39**	0.01	0.01	19.87	**20.25**
	32-16	3	16-8-4	20.80	**21.12**	28.39	**26.67**	0.01	0.01	20.13	**20.44**
	16-8		32-16-8	20.80	**21.10**	28.38	**26.65**	0.01	**0.01**	20.13	**20.42**

**Table 6:** RF results for viscosity and DYS values.

Metrics	MAE		RMSE		R-Squared		Logcosh	
	Train	Test	Train	Test	Train	Test	Train	Test
**Viscosity**	0.68	**1.23**	0.86	**1.54**	0.95	**0.80**	0.29	**0.73**
**DYS**	6.20	**8.97**	8.59	**12.11**	0.90	**0.76**	5.55	**8.32**

**Table 7:** NCART results for viscosity and DYS values.

Metrics	Parameter			MAE		RMSE		R-Squared		Logcosh	
	Tree	Layer	Select	Train	Test	Train	Test	Train	Test	Train	Test
**Viscosity**	2	2	4	0.22	**1.04**	0.38	**1.33**	0.98	**0.84**	0.06	**0.57**
	2	2	6	0.06	**1.20**	0.09	**1.58**	0.99	**0.79**	0.005	**0.73**
	6	2	6	0.05	**1.28**	0.07	**1.68**	0.99	**0.76**	0.003	**0.80**
**DYS**	6	2	6	0.62	**8.73**	1.26	**11.50**	0.99	**0.77**	0.31	**8.09**
	2	4	4	0.57	**9.65**	0.84	**13.42**	0.99	**0.66**	0.27	**9.00**
	6	4	6	0.73	**10.42**	1.32	**13.82**	0.99	**0.64**	0.39	**9.76**

**Table 8:** Best results obtained for viscosity and DYS parameters.

Model	MAE		RMSE		R-Squared		Logcosh	
	Train	Test	Train	Test	Train	Test	Train	Test
**Viscosity**								
NCART	0.22	**1.04**	0.38	**1.33**	0.98	**0.84**	0.06	**0.57**
RF	0.68	**1.23**	0.86	**1.54**	0.95	**0.80**	0.29	**0.73**
CNN	1.96	**2.40**	2.43	**2.76**	0.61	**0.28**	1.41	**1.78**
**DYS**								
NCART	0.62	**8.73**	1.26	**11.50**	0.99	**0.77**	0.31	**8.09**
RF	6.20	**8.97**	8.59	**12.11**	0.90	**0.76**	5.55	**8.32**
CNN	15.07	**19.02**	19.99	**23.00**	0.47	**0.01**	14.41	**18.33**

**Table 9:** Best results obtained for viscosity and DYS parameters using augmented data.

Model	MAE		RMSE		R-Squared		Logcosh	
	Train	Test	Train	Test	Train	Test	Train	Test
**Viscosity**								
**NCART**	0.22	**1.04**	0.38	**1.33**	0.98	**0.84**	0.06	**0.57**
**NCART-LLM**	0.37	**0.53**	0.58	**0.78**	0.97	**0.95**	0.11	**0.21**
**RF**	0.68	**1.23**	0.86	**1.54**	0.95	**0.80**	0.29	**0.73**
**RF-LLM**	0.78	**0.97**	0.97	**1.20**	0.93	**0.89**	0.35	**0.50**
**CNN**	1.96	**2.40**	2.43	**2.76**	0.61	**0.28**	1.41	**1.78**
**CNN-LLM**	0.70	**0.75**	0.97	**1.04**	0.93	**0.91**	0.32	**0.36**
**DYS**								
**NCART**	0.62	**8.73**	1.26	**11.50**	0.99	**0.77**	0.31	**8.09 **
**NCART-LLM**	1.11	**3.29**	1.66	**5.01**	0.99	**0.95**	0.65	**2.84**
**RF**	6.20	**8.97**	8.59	**12.11**	0.90	**0.76**	5.55	**8.32**
**RF-LLM**	5.30	**6.88**	7.12	**9.52**	0.93	**0.85**	4.66	**6.24**
**CNN**	15.07	**19.02**	19.99	**23.00**	0.47	**0.01**	14.41	**18.33**
**CNN-LLM**	2.54	**3.27**	4.57	**5.43**	0.97	**0.95**	2.01	**2.71**

## Data Availability

The original contributions presented in this study are included in the article. Further inquiries can be directed to the corresponding author.

## Abbreviations

The following abbreviations are used in this manuscript:

AI	Artificial intelligence
BMS	Ball measuring system
CNN	Convolutional Neural Network
DEG	Diethylene glycol
DEIPA	Diethanol isopropanol amine
DYS	Dynamic yield stress
E	Ethylene glycol
F	Final viscosity
LF	Linear Regression Function
LS	Lignosulphonates
MAE	Mean Absolute Error
MARS	Multivariate Adaptive Regression Splines
MARC	Multiple summation regression trees
M-TEA	Modified triethanolamine
NCART	Neural Classification and Regression Tree
GA	Grinding aids
PCE	Polycarboxylate Ether-Based Water-Reducing Admixture Superplasticizer
PF	Power Regression Function
RF	Random Forest
RMSE	Root Mean Square Error
TEA	Triethanolamine
TIPA	Triisopropanolamine
